# Predictors of receiving COVID-19 vaccine among adult population in Iran: an observational study

**DOI:** 10.1186/s12889-023-15409-0

**Published:** 2023-03-14

**Authors:** Hassan Soleimanpour, Ehsan Sarbazi, Elham Davtalab Esmaeili, Ahmad Mehri, Saber Ghaffari Fam, Hossein-Ali Nikbakht, Mohammad Saadati, Saman Sedighi, Mohebat Vali, Hosein Azizi

**Affiliations:** 1 Emergency Medicine Research Team, Tabriz University of Medical Sciences, Tabriz, Iran; 2 Student Research Committee, Tabriz University of Medical Sciences, Tabriz, Iran; 3 Road Traffic Injury Research Center, Tabriz University of Medical Sciences, Tabriz, Iran; 4 Department of Epidemiology, School of Public Health and Safety, Shahid Beheshti University of Medical Sciences, Tehran, Iran; 5 Department of Epidemiology, Hamadan University of Medical Sciences, Hamadan, Iran; 6 Social Determinants of Health Research Center, Health Research Institute, Department of Biostatistics & Epidemiology, School of Public Health, Babol University of Medical Sciences, Babol, Iran; 7 Department of Public Health, Khoy University of Medical Sciences, Khoy, Iran; 8 Department of Neurosurgery, Keck School of Medicine, University of Southern California, Los Angeles, CA USA; 9 Student Research Committee, Shiraz University of Medical Sciences, Shiraz, Iran; 10 Women’s Reproductive Health Research Center, Tabriz University of Medical Sciences, Tabriz, Iran

**Keywords:** Acceptance, COVID-19 vaccine, Epidemiology, Hesitancy, Intention, Iran, Vaccination, Pandemic, Web survey, Conspiracy, Trust, Willingness

## Abstract

**Background:**

Vaccination is one of the best ways to stop the transmission of coronavirus disease 2019 (COVID-19). In this regard, uunderstanding the features related to the intention of different populations to receive the COVID-19 vaccine is essential for an effective vaccination program. This study aimed to investigate the vaccination intention predictors in the general adult population of Iran.

**Methods:**

A cross-sectional, web-based survey was conducted on social networks, including Telegram, WhatsApp, LinkedIn, Instagram, and Facebook. Multinomial logistic regression models were used to investigate predictors associated with the intention to receive COVID-19 vaccines, including sociodemographic characteristics, trust, worry, sources of information, and conspiracy beliefs. The main outcomes included unwillingness, undecidedness, and intention to receive the COVID-19 vaccine.

**Results:**

Out of 780 respondents, 481 (61.6%) reported an intention to be vaccinated, 214 (27.4%) expressed their undecided status, and 85 (10.9%) reported unwillingness to receive any type of COVID-19 vaccine. A higher age (OR undecided = 0.97, 95% CI (0.96–0.99)), (OR unwilling = 0.97, 95% CI (0.95–0.99)); exposure with COVID-19 (OR unwilling = 0.82, 95% CI (0.76–0.89)), (OR undecided = 0.87, 95% CI (0.83–0.93)) were positively associated with vaccination intentions. No/low trust in vaccines, institutions, concerns about the future of the pandemic, and conspiracy beliefs were strongly and negatively associated with COVID-19 vaccination intentions.

**Conclusion:**

Most Iranians intended to get a COVID-19 vaccine. Higher vaccine acceptance needs to consider demographic features, exposure history, confidence in vaccines, trust in institutions, concerns, and conspiracy beliefs of people.

## Introduction

In March 2020, the World Health Organization (WHO) characterized coronavirus disease 2019 (COVID-19) as a pandemic, which led to interruptions in daily life such as closing businesses and schools and limiting social gatherings to prevent the spreading of the virus [1]. Globally, there have been more than 150 million cases, resulting in more than three million deaths [2]. Over five million infections were reported among the Iranian population, out of which, almost 123,000 patients died due to the severe symptoms of COVID-19 (9th October 2021) [3]. The COVID-19 illness spectrum is broad, ranging from asymptomatic infection to acute respiratory distress syndrome, culminating in death [4, 5].

The most important intervention against COVID-19 is believed to be mass vaccination, encouraging hundreds of organizations and institutions to strive for high-efficacy vaccine production [6, 7]. Dozens of vaccine development programs have been initiated in response to the pandemic [8]. Access to an effective vaccine can significantly aid in reducing virus transmission and hospitalization [9]. Among numerous recommendations regarding individual and social preventive measures (e.g., quarantine, using masks, physical distancing, hand washing, etc.), effective and safe COVID-19 vaccination is considered as the most efficient way to break the transmission cycle [10, 11].

One of the top ten global health challenges in 2019 was vaccine hesitancy [12]. Hesitation can seriously impact trust in vaccine by causing delays, refusals, and program problems in both research and delivery. On occasion, it may also cause disease epidemics to reoccur [13]. To achieve maximum immunization coverage, governments must improve public confidence, manage vaccine hesitancy, and create strategies for community involvement. [12] [15] [13].

Vaccine hesitancy is a complex and multifaceted issue that is influenced by a variety of emotional, cultural, social, spiritual, political, and other factors. Depending on the nation, the immunization, and the time of day, it may vary. There have been reports of low acceptance rates for the COVID-19 vaccination in the Middle East and other nations [14]. It is recommended to carry out extensive research to evaluate the degree of COVID-19 vaccination willingness [14].

The emerging of the disease, politicization of the vaccine, lack of vaccine selection and distrust in experts and governments have increased uncertainty about COVID-19 vaccination [3, 15, 16]. [4, 19–21] [17] vaccine distribution chains [3, 18] Lack of trust in vaccination and comes from less confidence about the vaccines’ effectiveness and side effects [17]. [22, 23].

[22] COVID-19 vaccination intentions have been surveyed and reported in previous studies [18, 19]. The proportion of willingness to undergo COVID-19 vaccination was 68.4% based on a meta-analysis [20]. [27] [28]About 20% of Iranian people showed hesitancy in getting COVID-19 vaccine [3]. Previous studies reported that sociodemographic variables such as male gender [21, 22], older [23], married [24], and highly educated individuals [25, 26], having a history of chronic disease [27], high income [28], health insurance [29], and high levels of self-efficacy [29] were associated with vaccine acceptance 37, 38.

[37] [38Accordingly, it is critical to understand the level of COVID-19 vaccine intention among different populations to have an efficient strategy for vaccination programs. This study aimed to investigate the factors associated with the intention to receive the COVID-19 vaccines among the general adult population in Iran.

## Methods

### Sampling and selection

This cross-sectional population-based study was conducted among general adult population in Iran from June to July 2021. Assuming the adult population of Iran to be 80,000,000 [30] with a vaccine acceptance of 78% [31] and margin of error of 2.9%, we calculated the sample size as 780.

### Inclusion and exclusion criteria

Inclusion criteria were being a resident in Iran at the time of completing the questionnaire, able to read and write in Persian, access to the internet via computer or smart phone, and age over 18 years. All mentally retarded individuals and those who received the COVID-19 vaccines were excluded from the study.

### Data collection

Due to the social distancing protocol, this survey study was conducted online in all provinces of Iran. We applied an anonymous online survey, which was answered voluntarily by the participants. After pre-testing, a web-based link was generated using the “Google forms” and the link was distributed on popular social networks, including the Instagram, WhatsApp, Telegram, LinkedIn, and Facebook and popular news websites. To advertise and circulate the survey, the research team shared the link with their network members. Network members and participants were also requested to distribute the survey invitation to all their contacts throughout the country. Volunteers in the study completed the checklist by clicking on the relevant link. Responses that were completed the same answer category repeatedly, blank forms, and repeat participation were removed. It took an average of 10–15 min for participants to complete the tool.

### Measurements

The questionnaire began with an information letter about the study purpose and how to answer the questions. Meanwhile, we obtained an informed consent from all participants in the study.

### Basic characteristics

The demographic characteristics included age, gender, occupation status, education (elementary, diploma, and college), economic status (1–10 scores), extent of exposure to COVID-19 (1–10 scores), personal, and family history of COVID-19 (yes, no). We also collected the information on history of chronic comorbidities such as diabetes, hypertension, cardiovascular disease, cancer, asthma or chronic obstructive pulmonary disease, obesity, hyperlipidemia, and kidney disease.

### Intention to get the vaccine

Intention to receive a COVID-19 vaccine was measured by the following question: “How likely do you think you are to get a COVID-19 vaccine if/when you are offered one?” (response options: yes, no, unsure or undecided) [32]. In the present study, hesitant people were defined as individuals who did not intend to be vaccinated and people who were unsure about getting vaccinated [33].

### Concerns, worrying about COVID-19 and conspiracy theory

To evaluate worrying, we used the following question derived from earlier investigation on the COVID-19 pandemic [34, 35]: “To what extent do you worry about the pandemic of COVID-19?” [42, 43]The participants answered using a 10-point scale (1 = Not at all, 10 = Extremely), with higher scores indicating higher levels of worrying about the COVID-19 pandemic. The conspiracy theory items were asked as yes/no questions based on the Salaam study which includes belief in conspiracy regarding origin of virus, controlling fertility, population numbers and human behaviors [22].

### Source of information and trust

Regarding the source of information on COVID-19, the participants choose the following sources in a 4-point scale (1 = No, 4 = A lot): radio, TV, overseas Iranian TV, newspapers, websites, social networks, medical doctors, health providers, scientific journals, and health ministry. Degree of trust was asked based on a Likert scale (0 = No, 1 = Low, 2 = Moderate, and 3 = High ) according to a study conducted in Ireland and the United Kingdom [36].

The checklist was revised and tested using 30 online questionnaires to determine the acceptability and clarity of the questions and confirm its face validity by experts. The responses obtained in the pilot study were not included in the final analysis.

Noteworthy, the reliability of the whole scale was calculated, and the Cronbach alpha was 0.92.

### Statistical method

To report descriptive statistics of quantitative variables, we utilized mean and standard deviation, and for qualitative variables, we used frequency and percentage. The Multinomial Logistic Regression (MLR) is intended to be used when you have a multiclass outcome variable (unwilling, undecided, intended) that does not have a natural order to it. The MLR model with a 95% confidence intervals (95% CI) was used for comparisons between unwilling and unsure versus intent participants. Then, variables with a significance level of less than 0.1 in the univariate analysis were entered into multivariate analysis. Statistical analysis was done using the STATA software version 15 (STATA Corp, College Station, TX, USA).

### Ethical considerations

Individuals who agreed to participate in the study entered the survey link anonymously. This study was conducted according to human ethics guidelines in medical sciences research in Iran. The researcher was responsible for the confidentiality of the forms and taking appropriate measures to prevent their publication. Moreover, the research was approved by the Ethical Committee of the Tabriz University of Medical Science, Tabriz, Iran (Approval ID: IR.TBZMED.REC.1400.719).

## Results

Overall, a total of 780 subjects participated in the study. The response rate for the survey was 69%. Among the respondents, 50.38% were male, 59.23% were in the age range of 18–34 years, and 84.4% had a university degree. Most respondents (61.6%) showed intention to be vaccinated. However, 27.4%.

and 10.9% of respondents were undecided and unwilling, respectively.

We compared the demographic features of those who intended to get a COVID-19 vaccine and those who were undecided or unwilling to be vaccinated. The results showed significant between-group differences, so that older individuals (P < 0.001) and those with a higher education (P = 0.04) were more probable to report willing to accept vaccine (Table 1).

In terms of average level of exposure to COVID-19, the group with intention to receive vaccine was significantly higher than the unwilling to accept vaccine and undecided groups (P < 0.001).

**Table 1:** Distribution of intention to receive COVID-19 vaccines by demographic characteristics and COVID-19 history (n = 780)

Variable		N (%)	Unwilling	Undecided	Intended	*P*-value
			N (%)	N (%)	N (%)	
Age*		780(100)	31.4 (1.1)	31.5 (0.8)	34.3 (0.4)	< 0.001^*^
Sex	Male	393 (50.3)	38 (9.7)	109 (27.7)	246 (62.6)	0.275
	Female	387 (49.6)	47 (12.1)	105 (27.1)	235 (60.7)	
Education**	Elementary	46 (5.9)	4 (6.6)	15 (32.6)	27 (58.7)	0.04
	Diploma	75 (9.6)	5 (6.6)	31(41.3)	39 (52.0)	
	College	659 (84.4)	76 (11.5)	168 (25.4)	415 (62.9)	
Economic status (1–10)*		780 (100)	4.49 (2.3)	4.58 (2.2)	4.53 (1.9)	0.887
COVID-19 disease	Yes	291 (37.3)	27 (9.2)	75 (25.7)	189 (64.9)	0.189
	No	489 (62.6)	58 (11.8)	139 (28.4)	292 (59.7)	
History of COVID-19 in family	Yes	393 (50.3)	37 (9.4)	108 (27.4)	248 (63.2)	0.173
	No	387 (49.6)	48 (12.4)	106 (27.3)	233 (60.2)	
Exposure to COVID-19*(1–10)		780 (100)	5.02 (3.0)	5.49 (2.7)	6.55 (2.7)	0.001^*^

P-value presented based on Chi-squared test, *Mean (standard deviation), P-value based on one way ANOVA, ** Chi-squared trend test

**Fig. 1:**
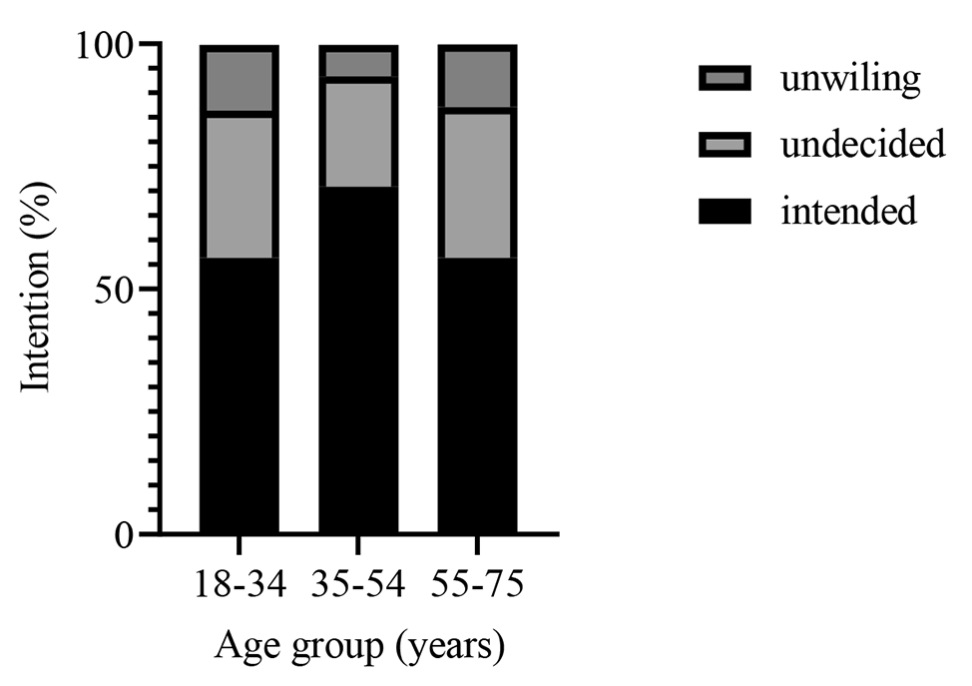
Distribution of intention to receive COVID-19 vaccines by age group

Figure 1 shows the distribution of intention to get vaccinated by age group. As can be seen, 56.4% of people with intention to get vaccinated were in the age ranges of 18–34 and ≥ 55 years. Nonetheless, among the undecided and unwilling individuals, the lowest rate was seen in the age range of 35–54 years. Also, the highest intention to receive vaccines was observed in the age group of 35–54 years.

### Sources of information and trust

**Fig. 2:**
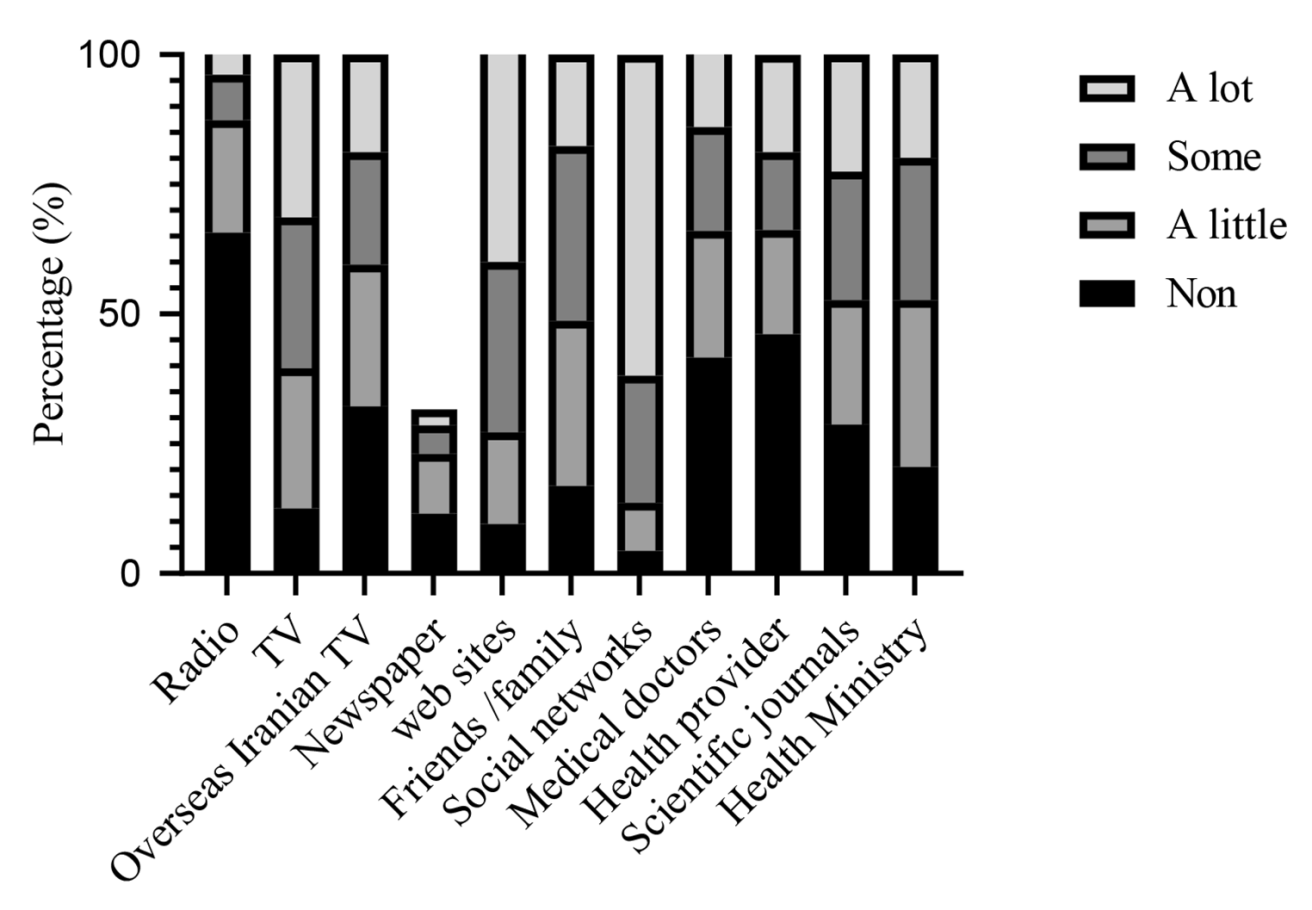
Various information sources to get COVID-19 news

For all sources of information, using of info was diverse between the three vaccine intention groups. Most respondents (63.0%) reported social networks (e.g., Facebook, Telegram, WhatsApp, Instagram) as a common source to receive COVID-19 information **[**Fig. 2**].** However, 3.8% of participants never used social networks to receive information. In addition, 80.0% of respondents stated that they never received information from the national newspapers (printed/online).

**Fig. 3:**
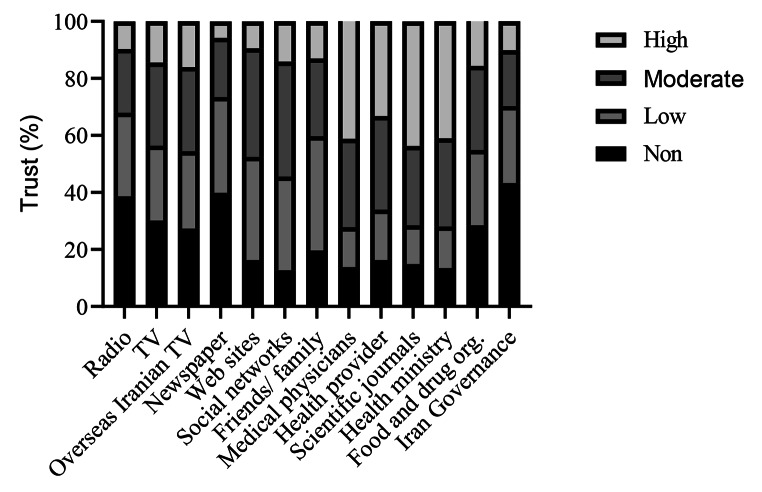
Levels of trust toward COVID-19 health information released by information sources

Although most participants used social media as a source of information, they showed less trust in this source of information. Furthermore, local authorities such as the Ministry of Health and Medical Education (MOHME) and MOHME’s spokesperson, food and drug administration, and public health providers were also relatively trusted. For all sources of information, the level of trust was significantly different between the three vaccine intention groups. The highest proportion of trust level (a lot) was reported to be from scientific journals (43.7%) and physicians (41.0%) [Fig. 3]. The level of trust in these sources was lower in the unsure and unwilling groups than in the intended group.

### Conspiracy beliefs about COVID-19 virus

As described in Tables 2 and 78.0% of respondents believed that the COVID-19 virus was man-made. Also, 48% of respondents believed that the COVID-19 virus was created to control population growth. Furthermore, 18% of the respondents believed that the COVID-19 vaccine was a way to implant microchips into the human body to control them. Moreover, 14% of participants believed that the COVID-19 vaccine would cause infertility. The conspiracy beliefs about vaccines significantly and negatively affected the Iranian adults’ intention to get vaccinated, which means that the more the Iranian adults believe in COVID-19-ralated conspiracy theories, the less they are inclined to get the vaccine.

**Table 2:** Conspiracy beliefs about COVID-19 virus and vaccines

Item	N (%)	Unwilling N (%)	Undecided N (%)	Intended N (%)	*P*-value
**Do you believe that …**					
… corona virus is man-made?	Yes (608.7)	75 (12.3)	181 (29.7)	352 (57.8)	< 0.001
	No (172.2)	10 (5.1)	13 (19.1)	129 (75.0)	
… corona virus was created to control population growth?	Yes (371.4)	52(14.0)	115 (31.0)	204 (54.9)	< 0.001
	No (409.5)	33 (8.0)	99 (24.2)	277 (67.7)	
… COVID-19 vaccination will be used to implant microchips into humans to control them?	Yes (138.1)	23 (16.6)	52 (37.6)	63 (45.6)	< 0.001
	No (642.8)	62 (9.6)	162 (25.2)	418 (65.1)	
… COVID-19 vaccination will lead to infertility?	Yes (106.1)	22 (20.7)	39 (36.7)	45 (42.4)	< 0.001
	No (674.8)	63 (9.3)	175 (25.9)	436 (64.6)	

P-value presented based on Chi-Squared test.

### Predictors of intention to receive a COVID-19 vaccine

Older age decreased the odds of receiving COVID-19 vaccines in unwilling (OR = 0.97; 95% CI = 0.95 to 0.99) and undecided subjects (OR = 0.97; 95% CI = 0.96 to 0.99) (Table 3). Respondents with more exposure to COVID-19 had less odds of being unwilling or undecided to receive COVID-19 vaccines (OR = 0.82; 95% CI = 0.76 to 0.89; OR = 0.87, 0.83 to 0.93). Individuals with a diploma degree of education had more odds of being undecided to get a COVID-19 vaccine than intended group (OR = 1.96, 95% CI = 1.18 to 3.25). There was no significant gender difference regarding intention to receive a COVID-19 vaccine.

Respondents who reported no/low trust in domestic vaccines showed significantly higher odds of being unwilling and undecided to receive COVID-19 vaccines compared to intended participants (OR = 5.42; 95% CI = 3.05 to 9.62; OR = 2.58, 95% CI = 1.84 to 3.62, respectively). No/low trust in imported vaccines had higher odds of being unwilling and undecided to receive COVID-19 vaccines (OR = 6.70; 95% CI = 4.07 to 11.05 and OR = 2.17, 95% CI = 1.53 to 3.06, respectively). No/low institutional trust associated with higher odds of unwilling and undecided intention (OR = 4.6; 95% CI = 2.70 to 7.84 and OR = 1.55, 95% CI = 1.12 to 2.15, respectively).

Regarding conspiracy theory, the belief in implanting microchips had higher odds among unwilling (OR = 2.46, 95% CI = 1.42 to 4.25) and undecided subjects (OR = 2.13; 95% CI = 1.41 to 3.21). Also, belief in infertility was higher among unwilling and undecided subjects than intended individuals (OR = 3.38, 95% CI = 1.91 to 6.01; OR = 2.16, 95% CI = 1.36 to 3.43, respectively). Conspiracy belief about man-made origin of the virus was associated with higher odds in unwilling and undecided individuals (OR = 2.75, 95% CI = 1.38 to 5.48; OR = 2.01, 95% CI = 1.32 to 3.06). Furthermore, the belief in population control increased the odds in unwilling and undecided individuals (OR = 2.13, 95% CI = 1.33 to 3.43; OR = 1.57, 95% CI = 1.14 to 2.18).

**Table 3:** Results of multinomial logistic regressions regarding the intention to get COVID-19 vaccines

Variable		Unwilling			Undecided		
		OR	95% CI	*P*-value	OR	95% CI	*P*-value
Age		0.97	0.95–0.99	0.029	0.97	0.96–0.99	0.002
Sex (Ref: male)	Female	1.29	0.81–2.05	0.27	1.01	0.73–1.39	0.959
Literacy (Ref: college)	Elementary	0.80	0.27–2.37	0.70	1.37	0.71–2.64	0.344
	Diploma	0.70	0.27–1.83	0.47	1.96	1.18–3.25	0.009
Economic status		0.99	0.88–1.11	0.883	1.01	0.28–0.61	0.732
Exposure to COVID-19		0.82	0.76–0.89	0.001	0.87	0.83–0.93	< 0.001
COVID-19 in family (Ref: No)	Yes	0.724	0.45–1.15	0.173	0.96	0.69–1.32	0.790
Trust in domestic vaccines (Ref: M&H)	Non/Low	5.42	3.05–9.62	0.001	2.58	1.84–3.62	< 0.001
Trust in imported vaccines (Ref: M&H)	Non/Low	6.70	4.07–11.05	0.001	2.17	1.53–3.06	< 0.001
Institutional trust (Ref: M&H)	Non/Low	4.60	2.70–7.84	0.001	1.55	1.12–2.15	0.007
Trust in social networks (Ref: M&H)	Non/Low	1.22	0.77–1.94	0.393	1.29	0.93–1.78	0.124
Trust in media (Ref: M&H)	Non/Low	1.77	0.99–3.15	0.053	1.03	0.72–1.47	0.872
Future concerns about the pandemic		0.82	0.76–0.8	0.001	1.02	0.97–1.07	0.412
Conspiracy belief: man-made (Ref: No)	Yes	2.75	1.38–5.48	0.004	2.01	1.32–3.06	< 0.001
Conspiracy belief: microchips (Ref: No)	Yes	2.46	1.42–4.25	0.001	2.13	1.41–3.21	< 0.001
Conspıracy belief: infertility (Ref: No)	Yes	3.38	1.91–6.01	0.001	2.16	1.36–3.43	< 0.001
Conspıracy belief: population control (Ref: No)	Yes	2.13	1.33–3.43	0.002	1.57	1.14–2.18	0.006

OR: Odds Ratios are adjusted for all variables, CI: confidence interval, Moderate/High(M&H), institutional trust (Iran Governance, ministry of health, fFood and dDrug Administration administration, health provider, and physicians), trust in government media (websites, radio, TV, and newspapers) * Belief that the COVID-19 virus is man-made, **Belief that the COVID-19 virus was created to implant a “microchip” in the human body, *** Belief that the COVID-19 vaccine causes infertility, **** Belief that the COVID-19 virus was created to control population growth, R square = 43

## Discussion

Hesitation to vaccination is a complex and dynamic social mindset. The unwillingness to get vaccinated is not just limited to the COVID-19 vaccine. However, evidence suggests that vaccine hesitancy has intensified in recent years [37–39].

The results of our study revealed that about 62% of participants were intended to receive the COVID-19 vaccines. However, about 11% of the participants were unwilling to participate in the global attempt to eradicate COVID-19, and about 27% of them were unsure about getting the COVID-19 vaccines.

In general, among the undecided and unwilling individuals, the lowest rate was observed in the age range of 35–54 years. This is in accordance with another study [40] that examined the desire for the COVID-19 vaccine and reported that older age was associated with a greater willingness to be vaccinated. This finding may reflect increased uptake associated with seasonal influenza vaccination in older age groups. It may also reflect an increased risk of morbidity and mortality from COVID-19 [41]. This is probably due to the greater risk of infection and developing a severe illness in older people [42]. Older age is one of the variables that not related to the perceived individual risk, but it is related to the fear of COVID-19, so that older people have more fear of COVID-19.

According to our results, a higher education level (college vs. diploma) increased the intent to get vaccinated. But in a similar study [43], a lower education was not statistically significant in predicting negative attitudes. In the mentioned study [43], the authors stated the non-significance of this relationship at the local government level, while this research measured education at the individual level and attitudes using a five-level scale. This may explain why no relationship was found between attitude and education. Perhaps one of the reasons for the higher willingness of educated people to get vaccinated is the level of awareness of these people, and it is also likely that these people trust science more and are less affected by anti-vaccine campaigns without research and scientific support. As a result, vaccination intentions differ depending on demographic factors, including age and educational attainment. This emphasizes the need for immunization programs that precisely target certain populations.

Our study did not observe any gender differences in the unwilling, undecided, and intended groups. But a previous study [44] stated that women were more willing than men to receive the COVID-19 vaccines. In general, there are mixed results regarding gender differences and vaccination reluctance in previous studies [43, 45, 46]. However, prior research indicated that while creating promotion tactics for vaccination programs, gender variations in attitudes and acceptability should be taken into account [44].

Our study did not observe any relationship between intention to receive COVID-19 vaccines and economic status. But another study [47] showed that respondents with an annual household income of less than $15,000 are less likely to receive this vaccine, which indicates the effect of economic status on the intention to receive COVID-19 vaccines. Perhaps part of the observed differences is related to the way the economic situation was measured in our study, which made the relationship of this variable invisible. Of course, we should also consider that the lower estimate may have happened in the economic situation in our study.

People who were exposed to COVID-19 or had a family history of COVID-19 were more likely to get the vaccine. However, the findings of the current study do not support the previous research [48]. This discrepancy could be attributed to differences in setting and population of two studies.

Solis Ares et al. studied the intention to receive the vaccine between June 2020 and January 2021 in 15 countries. The Low-To-Middle-Income Countries (LMICs) had an average acceptance rate of 80.3% overall, with Pakistan and Burkina Faso having the lowest acceptance rates at 66.5%. Additionally, LMICs had acceptance rates that were greater than those of the US (64.6%) and Russia (30.4%) [49]. Similarly, our study reported 62% acceptance rate for the COVID-19 vaccines, which is like other LMICs, though higher compared to reports from Russia.

Because of social media, the speed of global health exchange information has significantly increased, which leads to the circulation of misinformation on the platforms [15]. Most of the participants in our study used social media as a source of information about COVID-19, while 3% never used the social networks. To promote vaccination (especially COVID-19 vaccines), we should know whether people want to be vaccinated or not, understand the reason for their willingness or unwillingness, and recognize the most reliable sources of information in their decision-making. More people can now use the internet and social media in the LMIC with the wide availability of smartphones. Although this broad access can be a crucial component in vaccination decision-making, it can cause many challenges by broadcasting inaccurate and incomplete information, such as anti-vaccine messages.

In our study, people generally trusted scientific journals and physicians. It was reported in another review that health workers were the most reliable source of guidance on COVID-19 vaccines [26]. However, about 43% of respondents did not trust the Iranian government’s information about COVID-19. The lack of trust in Iranian vaccines was 38%, and 33% were not confident in the imported vaccines.

About 77% of the participants believed that the virus was manufactured, and 17% thought that the vaccine was a way to implant a microchip in the body. Also, 13% of participants believed that the vaccines could lead to infertility. Meanwhile, 65% and 47% of respondents believed that corona virus created to compete economically and control population growth, respectively.

There was less concern about the future of COVID-19 in the group of respondents who did not intend to receive the vaccine. Other studies have suggested that vaccine acceptance is related mainly to the interest in personal protection against COVID-19; this is while concerns about side effects are the most common reason for doubt [49]. Therefore, researchers and drug manufacturers should make the data on the COVID-19 vaccine as accessible as possible. Government should also be clear and sincere about COVID-19 vaccination programs and the availability of vaccines. Reporting adverse events and complications after immunization is a critical component of monitoring vaccination programs. The intensive media coverage may also deter people from being vaccinated, while it is essential to report the adverse effects. Therefore, the media must broadcast responsibly and provide clear and unbiased information to their audiences.

People (including medical professionals) who utilize the internet and social media should be aware that they are responsible for preventing incorrect sharing of content and avoid using language that may be misinterpreted, which might add to the suspicion for vaccine injection. Although vaccine distribution justice remains a significant challenge for LMICs, delays in delivering COVID-19 vaccines in these areas may also cause hesitation [45].

The main strength of this study was the relatively large number of respondents. However, this research offers helpful data regarding Iranian adults’ intentions about vaccines that might be used to develop future pandemic responses. Additionally, fresh information is made available to the public everyday throughout the epidemic, which may affect respondents’ impressions. So, it seems that careful design of targeted vaccination awareness campaigns for public decision-makers can effectively increase vaccination uptake among vaccine-hesitant people to achieve herd immunity in Iran.

### Limitations

However, the results should be interpreted with caution due to several limitations. First, the primary analyses were conducted in a cross-sectional manner. For sub-analysis of these significant categories, there was an insufficient number of minorities, older persons, or those with health conditions that put them at risk for severe COVID-19 illness. Other limitations of this study include self-report biases, potential impacts of unmeasured confounders, and biases from our sampling approach, which are all common in survey-based research.

Although we used random sampling method using social media to encourage the public to participate in the study, our study did not include individuals without a smartphone or people who decided not to respond online surveys. So, attention must be paid to any attempts to generalize small and specific subpopulations to the population level.

## Conclusion

In total, our findings showed that about 62% of people intended to get vaccinated. Our study also indicated why respondents intend or do not intend to receive the COVID-19 vaccine. Higher vaccine intention need to consider demographic characteristics, exposure history, trust in vaccines, trust in institution, concerns and conspiracy beliefs, which should be informed scientifically and clearly. Also, health professionals and scientific journals were the most reliable sources of information, and their role in people’s decisions should be encouraged. These results are crucial for guiding healthcare professionals’ and health officials’ adherence to the COVID-19 vaccination.

## Data Availability

The datasets used and/or analyzed during the present study are available from the corresponding author upon request.
